# Task Construal Influences Estimations of the Environment

**DOI:** 10.3389/fnhum.2021.625193

**Published:** 2021-06-10

**Authors:** Vjeran Keric, Natalie Sebanz

**Affiliations:** Department of Cognitive Science, Central European University, Budapest, Hungary

**Keywords:** energetic costs, effort, distance estimation, height estimation, visual perception

## Abstract

People’s characteristics can affect their perception of the physical environment, and the judgments and estimates they make about their surroundings. Estimates of the environment change based on observers’ metabolic state, physical properties, and the potential effort they would need to exert for a certain action. The functional role of such scaling is to provide agents with information on possible actions and their energetic costs. Combining actions with costs facilitates both higher-level planning (e.g., choosing an optimal running speed on a marathon) as well as planning on lower levels of an action hierarchy, such as determining the best movement trajectories for energy-efficient action. Recently, some of the findings on reported effects of effort on perception have been challenged as arising from task demands—participants guessing the purpose of the experimental manipulation and adjusting their estimates as a result. Arguably however, the failed replications used overly distracting cover stories which may have introduced task demands of their own, and masked other effects. The current study tested the generality of effects of potential effort on height and distance perception, employing effective yet not distracting cover stories. Four experiments attempted to identify conditions under which anticipated effort may systematically change perceptual estimates. Experiment 1 found that height estimates were not influenced by the effort required to place objects of different weights onto surfaces of varying heights. Experiments 2, 3 used two different effort manipulations (walking vs. hopping; and carrying an empty vs. a heavy backpack, respectively) and found that these did not influence estimates of distance (to be) traveled. Experiment 4 also used backpack weight to manipulate effort but critically, unlike Exp. 1–3 it did not employ a cover story and participants did not traverse distances after giving estimates. In contrast with the first three experiments, distances in the final experiment were estimated as longer when participants were encumbered by a backpack. Combined, these results suggest that the measured effects on the estimation of distance were due to how participants construed the task rather than being of a perceptual nature.

## Introduction

The way humans perceive, act, and think is shaped by properties of the body and its possibilities for interacting with the environment. Long before Embodied Cognition approaches became prominent (for reviews, see e.g., Wilson, 2002; Shapiro, 2011; Galetzka, 2017), Gibson (1979) argued that the visual system is geared toward perceiving properties of the world and objects within it that allow organisms to interact with the environment in a particular way (“affordances”). A roof affords hiding from the rain, a chair affords sitting, and a pencil affords grasping with a precision grip. The perception of these properties is based on the relations between a specific agent and the world; a steep cliff affords different actions to mountain goats and humans. People are adept at judging which actions are at their disposal. While Gibson focused on “direct perception,” a large number of studies suggests that perceiving affordances is tightly coupled with action execution (e.g., Craighero et al., 1996; Tucker and Ellis, 1998, 2001; Buccino et al., 2009; Cardellicchio et al., 2011; Janyan and Slavcheva, 2012).

There is an increasing amount of evidence suggesting that the way we perceive the environment is influenced by our capabilities. For example, the same object will be judged as closer if one has a tool extending one’s reach (Witt et al., 2005). Precise archers estimate targets as larger (Lee et al., 2012) compared to imprecise ones, proficient jumpers judge distances as shorter (Lessard et al., 2009) and participants trained in parkour see walls as shorter than those with little experience in it (Taylor et al., 2011). These results suggest that the properties and capabilities of our bodies are a “ruler” of sorts against which the environment is measured.

Why would this be the case? According to the interface theory of perception (Hoffman et al., 2015), natural selection has shaped perception so that it guides adaptive behavior. Relatedly, Proffitt (2006) and Proffitt and Linkenauger (2013) posit that a crucial role of perception is to inform efficient action. In this view, an action is “efficient” if it completes a task with the least energy expenditure. It is assumed that the perceptual system evolved in a way that gave our ancestors an advantage, and one of the greatest advantages of all is optimization of energy consumption. According to this view, an important role of perception is to integrate information and to provide an agent with a view of the world with all contextual considerations already factored in. The central prediction is that energetically expensive actions are coupled with percepts that overestimate the features of the environment that need to be overcome in order to execute these actions.

This prediction has been supported by a number of studies, mostly focusing on how metabolic states and energy expenditure influence perceptual judgments about distances and the steepness of surfaces. Slants of hills are judged as steeper when the participant making the judgment is encumbered by a heavy backpack, in poor physical shape, tired from previous physical activity or old rather than young (Proffitt et al., 1995, Proffitt et al., 2003; Bhalla and Proffitt, 1999). Hills that are very difficult to climb down from but manageable to climb up on seem steeper when viewed from the top as opposed to the bottom (Proffitt et al., 1995). Furthermore, if perception is tied to bodily states, metabolic changes should influence perceptual judgments. In a series of experiments, Schnall et al. (2010) gave one group of participants a caloric drink before they made slant judgments. Participants who consumed the caloric drink judged slopes as gentler compared to controls who drank a placebo. These findings were replicated for judgments of distance perception (Zadra et al., 2016).

While there is substantial evidence for energetic costs influencing estimates of distance and slope, including a recent meta-study confirming that effort influences distance estimates (Molto et al., 2020), others have criticized these findings, proposing that many of the described studies investigated biases in judgment rather than perception (Firestone and Scholl, 2016). For example, when estimating slants while carrying a heavy backpack, participants could have easily deduced why the backpack was introduced and then intentionally or unintentionally adjusted their judgment, so that the measured effect could have been due to task demands rather than increased effort biasing participants’ estimates. In order to test for this possibility, Durgin et al. (2009) conducted a study in which carrying the backpack was embedded in an elaborate story. Participants were fitted with electrodes around their ankles and were told that the backpack contains electromyography equipment needed to measure muscle activation. The cover story eliminated overestimation and participants who were convinced by it judged slopes to be equally steep as participants not carrying a backpack. This suggests that the effects in other studies could have been due to participants inferring the goal of the backpack manipulation. In a later study, Durgin et al. (2012) found that if participants carrying a heavy backpack were informed about the role of the backpack and asked to ignore it, they did not estimate the slant of a hill differently than control participants not carrying a backpack.

One criticism of the experiments by Durgin et al. (2009, 2012) is that they introduced task demands of their own. More specifically, in Durgin et al. (2009) the cover story included carrying a noisy backpack, which might have drawn participants’ attention to the backpack, while in Durgin et al. (2012) participants were explicitly told to ignore the backpack. It is possible that explicitly ignoring the weight of a backpack or attending to a noisy one interferes with the heuristic that scales distance and slope estimates with potential energy expenditure (Clore and Proffitt, 2016). Furthermore, the explicit instruction to ignore a heavy backpack might have biased participants’ estimates in the opposite direction (Proffit, 2013; Witt, 2011; Witt et al., 2016).

In the present study, we investigated whether effort influences perceptual estimates when effective cover stories are employed that are neither distracting nor overly salient. In four experiments we investigated whether effort influences estimates of height (Experiment 1) and distance (Experiments 2–4). In Experiment 1, participants were asked to place an object on a shelf, and then estimate its height. Effort was manipulated by varying the height of the shelf as well as the weight of the object. The aim was to test if previous findings of effort influencing estimates also hold for vertical distance. Experiment 2 tested whether effort influences distance estimates in a novel task where effort was manipulated by varying the difficulty of locomotion. After giving estimates about the distance of a target, participants moved to the target by either walking (“low effort” condition) or by hopping on one leg (“high effort” condition). The goal was both to conceptually replicate Proffitt et al. (2003), as well as to see if a different kind of physical effort can bring about the effect. Since neither Experiment 1 nor Experiment 2 provided evidence for effects of effort on perception, we conducted two further experiments that included conditions closer to the ones studied by Proffitt et al. (2003). In Experiment 3, effort was manipulated by carrying a light or heavy backpack, followed by estimates of the traversed distance. This experiment still did not show the expected effects of anticipated effort on perception. However, anticipated effort modulated distance judgments in Experiment 4, where the cover story was dropped. We discuss these findings with regard to the role of social influences on perceptual judgments and consider implications for ongoing debates on the cognitive penetrability of perception.

## Experiment 1: Lifting Heavy Objects

The aim of the first experiment was to provide a conceptual replication of previous findings showing that distance estimates are modulated by effort. Participants were asked to lift objects of different weight and place them on shelves of various heights. The prediction that effort should influence height estimates follows from previous research. If effort changes height estimates in a similar way as those of distance (Proffitt et al., 2003; Josa et al., 2019) and slant (Bhalla and Proffitt, 1999), lifting heavier weights should increase perceived height. Furthermore, it is possible that placing weights on higher surfaces, requiring more effort, would influence estimates more strongly than placing them on lower ones. The experiment employed both an explicit verbal measure and a non-verbal one in which participants were asked to mark the height of the surface on a schematic drawing representing all the possible heights. The reasoning behind this was that similar results across different measures would provide stronger evidence that effort influenced estimates. In order to ensure that potential differences in estimates were not due to task demands, a cover story was employed. A final consideration relates to anticipating to act rather than giving estimates *per se*. It has been suggested that the intention to execute an action may be a precondition for effort influencing estimates (Witt et al., 2004). Therefore, in this experiment participants knew they would execute the actions after having given their estimates.

### Materials and Methods

#### Participants

Nineteen right-handed participants (mean age = 26.76, *SD* = 2.56, 14 female) signed up for this study and received gift vouchers (1500 HUF) for their participation. All participants had normal or corrected-to-normal vision, were naive to the purpose of the study, signed a consent form before testing began and were debriefed at the conclusion of the experiment. The study was approved by the United Ethical Review Committee for Research in Psychology (EPKEB) and was conducted in accordance with the Declaration of Helsinki (1991).

#### Design and Apparatus

**FIGURE 1:**
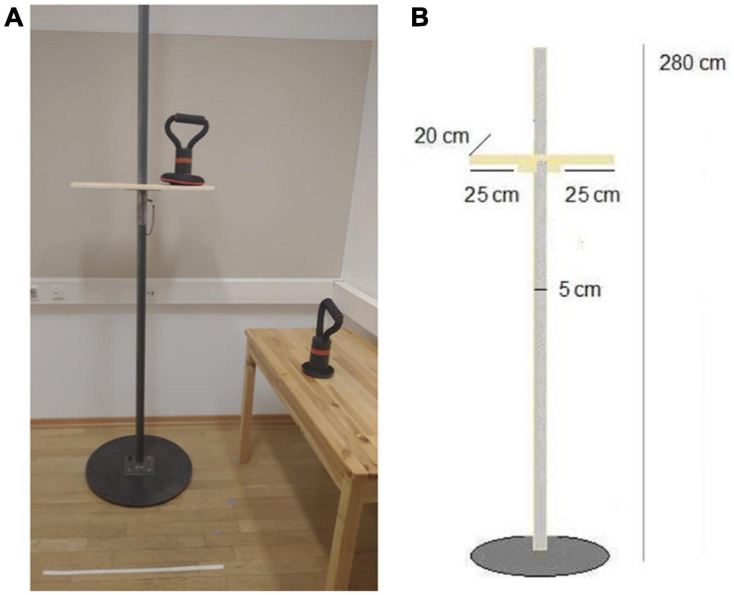
Experimental setup with two example weights (light and medium) marked with horizontal red stripes where participants were asked to place their thumb while grasping the weight **(A)**. Dimensions of the shelf and pole **(B)**.

The experiment manipulated effort by varying the weight of an object and the height of a shelf on which it had to be placed. The three weights (see Figure 1) used were (1) an empty base of an exercise weight, (2) the base with an added 1.7 kg, and (3) the base with an added 3.4 kg (coded as light, medium and heavy). A shelf of adjustable height (in 5 cm increments) was used for the height manipulation (see Figure 1). The heights were divided into three ranges: low, medium and high. This resulted in a 3 × 3 within-subject design with variables of Weight (light, medium and heavy) and Height (low, medium and high). Two dependent measurements were taken; verbal and non-verbal. Verbal estimates were reported in terms of shelf height in centimeters. For the non-verbal measure, participants marked the height of the shelf on a schematic representation of the pole that held the shelves.

#### Procedure

Before testing began, participants read an information sheet and signed consent forms. As part of the cover story, they were told that the apparatus would be used in future experiments and that the purpose of the present study was to test shelf stability under different weights and whether the shelves allowed for precise height estimates.

Participants were positioned in front of the apparatus so that the pole holding the shelves was aligned to their body midline. They were instructed to stand between 10 and 40 cm from the pole, depending on where they felt most comfortable to place objects on the shelf. Once a comfortable distance was determined, it was kept constant throughout the experiment. A trial started with the participant giving a verbal estimate of the height of the shelf and by marking the height on a line representing the height of the apparatus (shelf to line scale was 1:14). Participants then took a weight from a desk on their right-hand side, placed it on the shelf, lowered their hand in a resting position next to their body, and then returned the weight to the desk. In order to make lifting the weights more difficult, participants were instructed to grasp the body of the weight rather than the handle (see Figure 1). While the participant was returning the weight to its original place, the experimenter adjusted the shelf for the next trial. The experiment took 20–25 min to complete.

Before testing, participants went through a few (3–5) practice trials. Once they indicated that they understood the procedure, the testing phase consisting of 27 trials began. Trials were blocked so that each participant consecutively went through 9 trials per weight, and the blocks were counterbalanced across participants, creating 6 counterbalancing orders. Between blocks, the experimenter asked the participant to grasp the weight that will be used in the next block and lift it over their shoulder. This was done in order for the participants to get a sense of the weight, but they were told that the experimenter was making sure they were holding the new weight correctly. The height of the shelf was divided into three ranges; low (80–100 cm), medium (10–125 cm), and high (130–150 cm) with participants going through 9 trials in each range. The exact heights in each range and the presentation order were randomized. At the conclusion of testing, participants’ maximum reaching height was measured; they were asked to stand so that their toes were touching the base of the pole and to place their palm on the shelf. To check if participants guessed the purpose of the weight manipulation, they were asked whether they had thought about why we used different weights. This was followed by a debriefing where the purpose of the study and the weight manipulation were explained. Finally, participants were asked if during testing it had occurred to them that the weights were intended to influence their height estimates. If they answered affirmatively to the question or mentioned the relationship between weights and height estimates in the previous, open-ended question, they were excluded from the analysis.

### Results

As shelf heights were selected randomly from predefined ranges, a preliminary analysis (one-way ANOVA) was run to confirm that there were no differences in assigned heights between the three weight conditions (*p* = 0.994), counterbalancing groupings (*p* = 0.997) or individual participants (*p* = 0.999). One participant correctly guessed the purpose of the study and was excluded from the analysis.

#### Verbal Responses

**FIGURE 2:**
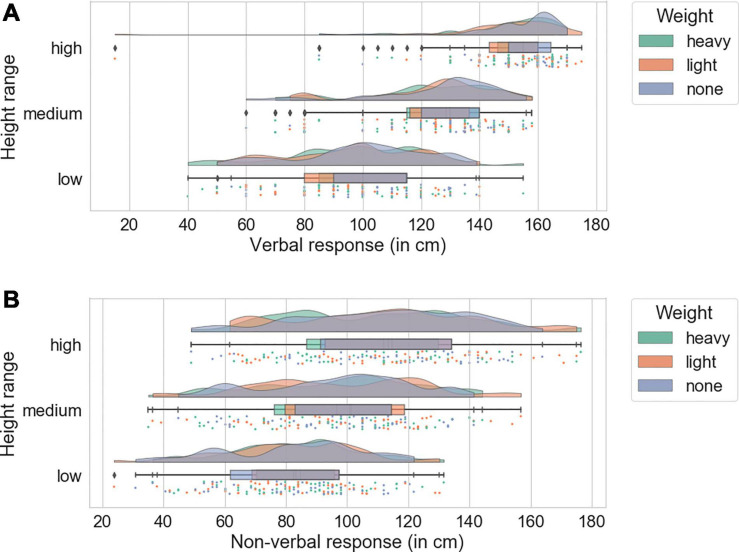
Participants’ verbal **(A)** and non-verbal responses **(B)** in Experiment 1 Superimposed distributions show responses in different weight conditions under which individual data points are vertically jittered. Boxes indicate interquartile range with median, whiskers show 1.5 interquartile range (modified from Allen et al., 2019).

Correlational analysis suggested a good overall performance on the task (see Supplementary Materials). A 3 × 3 × 6 mixed ANOVA was conducted with the within-subject factors of Weight (light, medium, heavy) and Height (low, medium, high) and counterbalancing order as a between-subject factor. The counterbalancing order was included in the analysis in order to control for carry-over effects. Previous studies showed that fatigue can influence estimates (Bhalla and Proffitt, 1999; Hunt et al., 2017) which could have created a situation in which participants’ estimates were influenced by a previous block of trials (e.g., overestimating heights in a “light” condition due to fatigue from previously completing “heavy” trials). Participants’ maximum reach was mean-centered and included in the analysis as a covariate. As Mauchly’s test revealed that a violation of the sphericity assumption occurred [χ^2^(2) = 6.86, *p* < 0.05], degrees of freedom were corrected using Greenhouse-Geisser estimates of sphericity (ε = 0.668). The results showed an expected main effect of height [*F*_(__1.34, 14.7)_ = 30.06, *p* < 0.001, η^2^*_*p*_* = 0.732] and no significant effect of weight or interactions (*p*s > 0.05). Counterbalancing order did not have a significant effect or significant interactions (*p*s > 0.05) and maximum reach was not a significant covariate (*p* > 0.05). For the distribution of verbal responses, see Figure 2A.

#### Non-verbal Responses

Correlational analysis suggested good performance, while also indicating that the non-verbal task was possibly more difficult than the verbal one (see Supplementary Materials). A 3 × 3 × 6 mixed ANOVA was performed with the within-subject factors of Weight (light, medium, heavy) and Height (low, medium, high), the between-subject factor of counterbalancing order, and maximum reaching height as a covariate. Greenhouse-Geisser correction was applied (ε = 0.719) because Mauchly’s test revealed that the sphericity assumption was violated [χ^2^(2) = 7.91, *p* < 0.05]. The results showed a significant main effect of height [*F*_(__1.44, 14.29)_ = 5.6, *p* < 0.05, η^2^_*p*_ = 0.338] and no other significant main effects or interactions (all ps > 0.05). For the distribution of non-verbal responses, see Figure 2B.

## Additional Analyses

In order to test whether the data support the null hypothesis that weight does not influence judgment estimates, the data were broken down by weight and a series of Bayesian paired-samples *t*-tests were conducted using a built-in function of JASP. In all tests, H_0_ stated that the effect size is δ = 0 while H_1_ assigned effect size a Cauchy prior centered on 0 with the interquartile range of *r* = 0.707. For verbal responses, comparing the influence of heavy and medium weight on participants’ estimates showed strong support for the null hypothesis (BF_01_ = 11.32). Comparisons between heavy and light weight received moderate support (BF_01_ = 7.82) as did those of light and medium weight (BF_01_ = 7.26). For non-verbal responses, the null hypothesis was strongly supported in all three comparisons showing BF_01_ ≈ 11 (see Supplementary Material for a more detailed analysis).

### Discussion

Manipulating effort did not seem to influence participants’ height estimates, regardless of the height range of the shelf they placed the objects on. For both verbal and non-verbal estimates, this conclusion is further supported by supplementary Bayesian analyses showing the data moderately to-strongly favor the null hypothesis with Bayes factors in favor of H_0_ ranging from BF_01_ ≈ 7 for verbal estimates to BF_01_ ≈ 11 for non-verbal ones. One possibility could be that the heights were too easy to judge. Perhaps computing effort may not have been necessary for estimations because participants were using their own height and reach to judge height. However, looking at the standard deviations in each condition (see Supplementary Table 1) and taking into account that participants misjudged the actual shelf height by 10 cm in verbal responses (M_actual_ = 114, M_estimated_ = 124.8), this does not seem very likely.

One open question is whether the effort manipulation should have been implemented taking into account participants’ physical characteristics. In the current study all participants, regardless of height, weight or fitness lifted the same weights. Most reported fatigue during the experiment or the debriefing but several said they thought the weights were meant to make it easier to place the weight holder on the shelf. While based on these reports it seems unlikely that the task was not sufficiently effortful, without adjusting the weights for each participant this possibility cannot be completely discounted.

Finally, it is possible that the results speak in favor of perceived task demands rather than effort influencing estimates. The cover story could have eliminated otherwise more obvious task demands, leading to physical effort having no effect on perceptual estimates of height. Taken together, the results of Experiment 1 did not provide evidence for the claim that energy expenditure influences estimates with regard to judgments of height, contrary to previous work reporting effects of effort on judgments of distance (Proffitt, 2006; Proffitt and Linkenauger, 2013).

## Experiment 2: Walking and Hopping Across Distances

This experiment followed a similar logic as Experiment 1, with two key differences. Firstly, instead of estimating heights, participants were asked to estimate distances. The reasoning was that previous studies have established that effort influences distance estimates whereas there is no available data on whether this is the case for heights. Secondly, instead of varying weight, the effort manipulation was operationalized by asking participants to cross distances by walking vs. hopping on one leg. Since effects of weight on distance estimates have been reported, we wanted to test whether other effort manipulations would yield similar effects. The main prediction was that the additional effort involved in hopping to a certain location compared to walking would increase estimates of its distance.

### Materials and Methods

#### Participants

Eighteen right-handed participants (mean age = 25.53, *SD* = 6.68, 13 female) signed up for this study and received gift vouchers (1500 HUF) for their participation. All participants had normal or corrected-to-normal vision, were naive to the purpose of the study, signed a consent form before testing began and were debriefed at the conclusion of the experiment.

#### Design

The experiment used a within-subject design. Participants traversed fourteen distances (1 m apart) while effort was manipulated by means of locomotion (walking vs. hopping). Dependent measures were participants’ verbal estimates of distance and the time it took for them to traverse a certain distance.

#### Apparatus and Procedure

**FIGURE 3:**
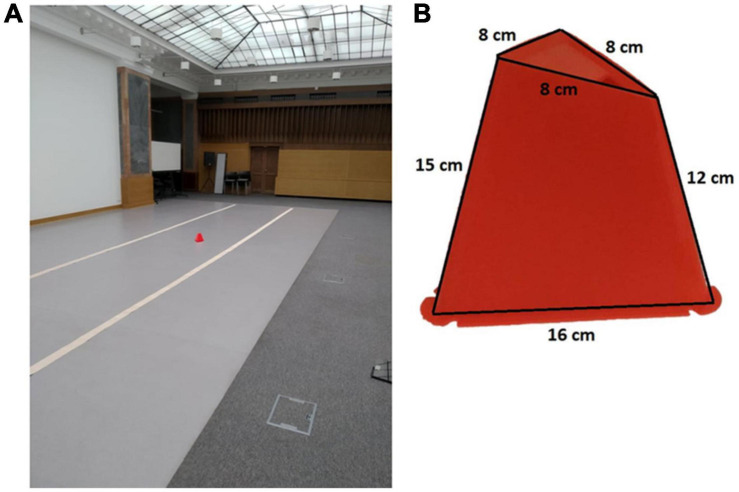
Setup in Experiments 2, 3 with the cone placed at 10 m from the participant **(A)**. Dimensions of the target cone. Dimensions of the target cone **(B)**.

The study was run in a large hall with a 14 m long and 1.5 m wide cardboard track marking the testing area (see Figure 3A). Participants read an information sheet, signed consent forms and the experimenter measured their height. The cover story was that the study is testing the relationship between the ability to balance one’s body and objects in one’s hand. The name of the study (“Balancing act”) served as an initial misdirection to the purpose of the experiment. To reinforce the story, before testing participants were asked to balance on one foot with their eyes closed while touching their nose with their index finger and then to walk along the track while balancing a ping-pong ball on a table tennis racket. Following the balancing tasks the experimenter said that the next part of the experiment is about maintaining body balance while walking and hopping and that the last part will return to balancing objects.

A trial began with the participant standing with their back turned to the track. The experimenter placed a cone (see Figure 2B) on the track (between 1 and 14 m away from the participant) and asked the participant to turn around, give a verbal estimate of the cone’s distance and then move to it and touch it with their dominant hand. The time from when the participant started moving to when they touched the cone was measured. After touching the cone, the participant walked back to the beginning of the track and remained with their back turned while the experimenter set the cone at the next distance. Each participant went through a “walking” and a “hopping” block with 14 trials per block. In “hopping” trials, participants’ legs were positioned such that the ankle of one leg was touching the knee of the other, while in “walking” trials they were instructed to move to the cone at a comfortable pace. Before testing, there were 4 practice trials (2 of each movement type). The order of distance presentation was random and the blocks were counterbalanced across participants. The experiment took between 25 and 35 min to complete. At the conclusion of testing, participants were informed that the study was not about testing balance and prompted to say what they thought the study was about. Finally, the aim of the study and the purpose of the manipulations was explained and they were asked if it had occurred to them during testing that hopping was intended to increase their distance estimates. Only participants who did not answer affirmatively to this question and had not guessed the purpose of the study during the open-ended question were included in the analysis.

### Results

One participant correctly guessed the purpose of the study and was excluded from the analysis, resulting in *N* = 17.

#### Verbal Responses

**FIGURE 4:**
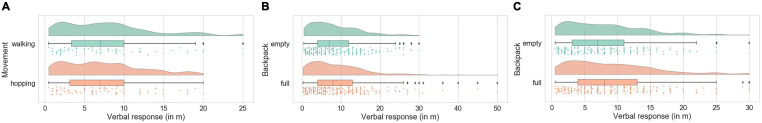
Participants’ distance estimates in Experiment 2 **(A)**, 3 **(B)**, and 4 **(C)**. Distributions show responses in different effort conditions under which individual data points are vertically jittered. Boxes indicate interquartile range with median, whiskers show 1.5 interquartile range (modified from Allen et al., 2019).

Correlational analysis suggested good overall performance on the task (see Supplementary Materials). A paired samples *t*-test revealed no significant difference between estimates in the hopping and walking conditions (*p* = 0.185). To test for order effects and the possibility that estimates were influenced by effort only at certain distances, a 2 × 14 × 2 mixed ANOVA was performed with the within-subject factors of movement (hopping, walking) and distance (1–14 m) and the between subject factor of counterbalancing order (walking first/hopping first). Participants’ height was mean-centered and included in the analysis as a covariate. Greenhouse-Geisser correction was applied because Mauchly’s test indicated a violation of the sphericity assumption [χ^2^(90) = 257.02, *p* < 0.001] for the main effect of distance [*F*_(__12.03, 28.363)_ = 98.88, *p* < 0.001, η^2^*_*p*_* = 0.876]. The results showed no other significant main effects or interactions (all *p*s > 0.05). For the distribution of estimates, see Figure 4A.

#### Movement Time Analysis

A 2 × 14 × 2 mixed ANOVA was performed with the within-subject factors of movement (hopping, walking) and distance (1–14 m) and the between subject factor of counterbalancing order (walking first/hopping first). Participants’ height was mean-centered and included in the analysis as a covariate. Mauchly’s test was significant for distance [χ^2^(90) = 232.96, *p* < 0.001]. The effect persisted after applying Greenhouse-Geisser correction [*F*_(__2.25, 33.88)_ = 131.44, *p* < 0.001, η^2^*_*p*_* = 0.898]. The main effect of movement was also significant [*F*_(__1,__16__)_ = 17.67, *p* < 0.001, η^2^*_*p*_* = 0.522] with participants moving faster in the hopping (M_hopping_ = 5.13 s, *SD* = 2.52) than in the walking condition (M_walking_ = 5.79 s, *SD* = 2.54). No other main effect or interaction reached significance.

## Additional Analyses

To establish if the data supports that the hopping and walking did not influence distance estimates differently, a Bayesian paired samples *t*-test was performed. As in the analysis in Experiment 1, the prior was Cauchy (0, 0.707). The analysis revealed weak evidence that the data support the null hypothesis BF_01_ = 1.36).

### Discussion

The results of Experiment 2 did not confirm our predictions. There was no significant main effect of the effort manipulation nor did it interact with distance. Participants’ reports during the experiment suggested that the effort manipulation was successful. At debriefing they overwhelmingly confirmed that it was more difficult to hop than to walk. Furthermore, during the procedure four participants asked if they could change the leg they were hopping on and an additional five asked how many trials were left because they were becoming tired.

Given that the results were non-significant and that the Bayesian analysis suggests that support for the null hypothesis was only anecdotal it is somewhat difficult to draw firm conclusions. It could have been the case that, apart from visual cues and effort, time needed to traverse the distances also served as a cue for estimation. If this was the case, then it is difficult to tell if the effort manipulation was successful since participants were faster in the hopping than in the walking trials. It is possible that effort increased distance estimates but that this effect was negated because traversing distances faster in the hopping condition made them appear shorter. A follow-up study controlling for time it takes to cross each distance could help disambiguate between these possibilities. Finally, as was the case in the previous experiment, it is possible that the cover story kept participants from perceiving task demands that could otherwise have affected distance estimates.

## Experiment 3: Carrying Weight Across Distances

Given that neither Experiment 1 nor Experiment 2 found effects of effort on perceptual estimates, the aim of the third experiment was to replicate previous findings using a more well-established paradigm (Proffitt et al., 2003). Furthermore, in Experiment 2, effort was manipulated by using different types of movement which left open the possibility that the faster speed in the more effortful condition mitigated a potential bias arising from increased effort. Experiment 3 followed the procedure by Proffitt et al. (2003) by manipulating effort by having participants carry a heavy or light backpack and used the same dependent measure (distance estimates). Unlike in the study by Proffitt et al., participants were instructed to walk to the target cones after giving estimates.

### Materials and Methods

#### Participants

Twenty participants (18 right-handed, mean age = 26.15, *SD* = 4.29, 11 female) signed up for this study and received gift vouchers (1500 HUF) for their participation. All participants had normal or corrected-to-normal vision, were naive to the purpose of the study, signed a consent form before testing began and were debriefed at the conclusion of the experiment.

#### Design, Apparatus, and Procedure

The design, apparatus and procedure were almost identical to Experiment 2. The design differed with respect to how the effort manipulation was implemented: instead of walking or hopping, participants walked while fitted with a heavy or empty backpack. As in previous experiments, participants read an information sheet, signed consent forms and the experimenter measured their height and weight before testing. The weight of the backpack was individually adjusted so that it was 20% (± 1.5 kg) of participants’ body weight. The cover story was that the study aims to investigate preferred walking speeds while carrying different items.

### Results

Two participants correctly guessed the purpose of the study and were excluded from the analysis. An additional participant was excluded due to making attempts to measure the length of the track while walking.

#### Verbal Responses

Correlational analysis suggested good overall performance on the task (see Supplementary Materials). A paired samples *t*-test revealed no significant differences in estimates in the two backpack conditions (*p* = 0.885). To test for the possibility that there was an effect of effort only at certain distances, an additional analysis was performed. The data was analyzed with a 2 × 14 × 2 mixed ANOVA with the within-subject factors of backpack (empty, full) and distance (1–14) and counterbalancing order (empty backpack first, full backpack first) as a between-subject factor. Participants’ height was mean-centered and included in the analysis as a covariate. Mauchly’s test of sphericity revealed a sphericity violation for distance [χ^2^(90) = 318.125, *p* < 0.001]. After applying Greenhouse-Geisser correction, the main effect of distance remained significant [*F*_(__1.39, 19.47)_ = 57.94, *p* < 0.001, η^2^_*p*_ = 0.805]. There were no other significant main effects or interactions. For the distribution of estimates, see Figure 4B.

#### Movement Time Analysis

A 2 × 14 × 2 mixed ANOVA was performed on time participants took to reach the target with the within-subject factors of backpack (empty, full) and distance (1–14 m) and the between-subject factor of counterbalancing order (empty backpack first/full backpack first). Participants’ height was mean-centered and included in the analysis as a covariate. Mauchly’s test indicated a violation of the sphericity assumption [χ^2^(90) = 148.8, *p* < 0.001] so degrees of freedom were adjusted using the Greenhouse-Geisser correction (ε = 0.312). The main effect of distance was significant, with longer distances leading to longer movement times [*F*_(__4.05, 56.76)_ = 639.23, *p* < 0.001, η^2^_*p*_ = 0.979]. Furthermore, there was a significant main effect of backpack weight [*F*_(__1, 1__6__)_ = 17.4, *p* < 0.001, η^2^*_*p*_* = 0.554], with participants walking faster when the backpack was empty (*M* = 6.12 s, *SD* = 0.58) as opposed to full (*M* = 6.5 s, *SD* = 0.45).

## Additional Analyses

A Bayesian equivalent of a paired-samples *t*-test was conducted using a built-in function in JASP. The analysis used a Cauchy prior (0, 0.707). Results suggested moderate support for the null hypothesis (BF_01_ = 3.88).

### Discussion

During debriefing participants reported that the backpack was heavy and that they needed to put in more effort to walk in the full backpack condition. Nevertheless, the findings did not show an effect of effort on distance estimates. The lack of a main effect of backpack was unexpected, especially given that the procedure closely resembled paradigms where such a manipulation modulated distance and slant estimates (Bhalla and Proffitt, 1999; Proffitt et al., 2003). Furthermore, a Bayesian paired-samples *t*-test showed that the data provide moderate support that there was no difference in estimates between the conditions. Interestingly, participants walked slower in the full than the empty backpack condition but their estimates did not differ. This speaks against our speculation about the results in Experiment 2, where we suggested that traversing distances faster in effortful conditions might have mitigated the effect of increased effort on distance estimation, and raises the question of which aspects of prior studies were missing from Experiment 3 to replicate prior results.

## Experiment 4: Standing in Place With a Heavy or Empty Backpack

In light of the series of null results in the previous experiments, the aim of Experiment 4 was to try to more closely replicate the original findings showing that carrying weight influences distance estimates. As was the case in earlier studies (e.g., Bhalla and Proffitt, 1999; Proffitt et al., 2003) participants were standing in place when giving estimates, no cover story was employed and the effort manipulation was implemented with a heavy or light backpack.

### Materials and Methods

#### Participants

Twenty participants (all right-handed, mean age = 24.18, *SD* = 2.8, X, 6 female) signed up for this study and received gift vouchers (1500 HUF) for their participation. All participants had normal or corrected-to-normal vision, were naive to the purpose of the study, signed a consent form before testing began and were debriefed at the conclusion of the experiment.

#### Design, Apparatus and Procedure

The design, apparatus and procedure were almost identical to Experiment 3. However, the procedure differed in two key aspects. After giving estimates, participants were not asked to walk to the target cone. Instead, they turned away from the track and a new trial began with placing the cone at a different distance. Secondly, unlike in the previous experiments, there was no cover story. Participants were not told anything about the purpose of the backpack nor how it related to their estimates. After the experiment, participants were asked an open-ended question about their opinion on what the experiment was about. This was done to see if they were aware that the backpack weight manipulation was intended to influence their distance estimates.

### Results

All participants except one correctly guessed the purpose of the weight manipulation. More precisely, when asked “what the experiment was about,” they answered that the purpose of the weight was to make them judge the target cone as being farther away. While explanations for the underlying mechanism differed across participants (e.g., two reported the backpack was intended to make thinking more difficult which would lead to higher estimates), all but one guessed the purpose of the manipulation. The participant who did not correctly guess the purpose of the manipulation was excluded on different grounds. During debriefing this participant reported that (s)he gave completely random estimates so that they can complete the experiment as quickly as possible. This was reflected in the data as their mean estimated distance was 167 m while the actual mean distance across the trials was 7.5 m. Correlational analysis suggested good overall performance on the task (see Supplementary Materials). A paired-samples *t*-test showed a significant difference between the full and empty backpack conditions [*t*_(__18)_ = 2.66, *p* = 0.016, d = 0.61)]. For the distribution of estimates, see Figure 4C.

## Additional Analyses

A Bayesian equivalent of a paired-samples *t*-test was conducted using a built-in function in JASP. The analysis used a Cauchy prior (0, 0.707). Results suggested moderate support for the alternative hypothesis (BF_10_ = 3.53).

### Discussion

The results of Experiment 4 suggest that potential effort of walking across a distance with a heavy backpack increased participants’ estimates of that distance. Given that the two main differences from the previous experiment are that participants stood in place and that there was no cover story, each of these could have made a difference. In particular, it could be argued that in Experiments 2, 3, participants traversed distances and that this allowed for better estimates. In such a case, moving across the distance would be a better basis for estimation and possibly make computations of effort unnecessary. However, this possibility seems unlikely, given that the additional analyses from the previous two experiments did not show any improvement in estimates as trials progressed (see Supplementary Materials). The more plausible explanation is that the results in Experiment 4 were due to perceived task demands (Durgin et al., 2012; Firestone, 2013; Firestone and Scholl, 2014**).** Removing the cover story made the purpose of the experiment apparent to the participants, which likely influenced their estimates.

## Summary and Conclusion

In the present study, we investigated whether effort influences perceptual estimates when the cover stories employed are both effective and not distracting. Four experiments were conducted to test how widely and robustly effort influences perceptual judgments of the environment.

Experiment 1 utilized a novel task in which participants estimated height while handling objects of different weights. Experiments 2, 3 investigated whether effortful locomotion and carrying weight increases distance estimates. Several key differences between these experiments and previous studies that found evidence for effort influencing estimates should be pointed out. Firstly, Experiment 1 used estimation of height rather than slope or distance as a dependent variable. The lack of significant effects of effort on estimates of height could be due to employing a successful cover story. However, it is worth noting that physical effort (in terms of height and weight of objects) was not controlled across participants. A potential follow-up would be to scale weights and shelf heights for each participant based on their height, reach, and fitness and repeating the experiment with and without a convincing cover story. Secondly, in Experiments 2, 3, participants traversed the judged distances while in previous studies they made estimates while standing in place (Proffitt et al., 1995, Proffitt et al., 2003; Bhalla and Proffitt, 1999; Meagher and Marsh, 2014; Josa et al., 2019). It might be the case that walking across the distances informed estimates better than effort which was consequentially disregarded. However, this seems unlikely for two reasons. On the one hand, additional analyses showed that participants’ performance did not improve in later trials compared to earlier ones. On the other hand, looking at the mean estimates of participants in each of the experiments, there seems to be no systematic indication that traversing the distance improved estimates or that the precision of the estimate was related to effort^1^.

Given the results of Experiment 4, where effort seemed to affect distance estimates in the absence of a cover story, we need to consider the possibility that the effort manipulations in Experiments 1–3 did not influence estimates due to the effectiveness of the employed cover stories. The rationale behind employing elaborate cover stories was based on critiques of previous findings proposing that estimates could have been influenced by perceived task demands rather than increase in effort (Durgin et al., 2012; Firestone, 2013; Firestone and Scholl, 2014). For example, Durgin et al. (2009) found that slants appeared steeper only to participants who guessed that they were fitted with a heavy backpack in order to manipulate their estimates. Taking into account criticism of Durgin et al. (2009, 2012) stories as being intrusive or biasing participants in the other direction (Proffit, 2013; Witt et al., 2016), the cover stories employed here were more subtle. Considering that our experiments used cover stories successfully, the possibility that findings of some of the previous studies are due to task demands rather than effort cannot be discounted. This possibility is supported by the fact that Experiment 4 did not use a cover story and the effect of effort was significant. Unfortunately, due to the low number of participants excluded based on correctly assessing the aim of the experiments (four across Experiments 1–3), statistical analysis of their estimates would not be informative. Taking the results of the four experiments together, they provide little evidence that effort influences the way physical properties of the world are estimated. Furthermore, Bayesian analyses suggest that evidence moderately leans toward effort having no effect when it comes to estimates, at least in Experiments 1, 3.

It should be pointed out that the literature suggesting that energy expenditure influences estimation is much broader and not all of the evidence relies on cover stories. Direct energy manipulations such as consummation of a caloric drink (Schnall et al., 2010; Zadra et al., 2016) or action-based measures or perceptual matching (e.g., Witt et al., 2004) might be more robust and independent from task demands. The same could be true for studies manipulating energy expenditure and visual flow (Proffitt et al., 2003; Experiments 2, 3; Zadra and Proffitt, 2016, Experiment 3). These (and other) studies provide converging evidence for effort influencing perception.

However, a growing number of experiments has put even this evidence into question. For example, Woods et al. (2009) failed to replicate effort-based effects using both verbal and action-based measures. Similarly, Shaffer et al. (2013) showed no effect of glucose on participants’ slant estimates. In that experiment, participants who consumed a placebo but believed it was a caloric drink reported the hill as less steep than those consuming a caloric drink while blind to the experimental manipulation. Taken together, studies by Durgin et al. (2009, 2012) and Shaffer et al. (2013) suggest that many of the effects interpreted as effort affecting perception may be a product of task demands. The present findings do not directly address the debate concerning energetic effects and certainly do not warrant the conclusion that all reported effects of effort were due to task demands. However, they provide evidence that estimates can be affected by how a task is framed or construed and should prompt further investigation to dissociate effects of effort from effects of task construal (which are interesting in their own right as a reflection of social influence).

A broader question our results touch upon is whether perception is susceptible to top down influences. There is a growing literature suggesting that perception is permeable, with inputs from memory, emotions and action seeping in. For example, language knowledge seems to influence color discrimination (Winawer et al., 2007), knowledge of object colors influences perception of grayscale objects (Witzel et al., 2011), desirable objects are judged as closer (Balcetis and Dunning, 2010) and the room participants are sitting in is judged as darker after reading about an immoral compared to a moral act (Banerjee et al., 2012). Following this line of reasoning, the results of the current experiments could suggest that how a situation is framed directly influences perception. On the other hand, serious arguments have been raised against top-down influences on perception (Firestone and Scholl, 2016). A problem specific to manipulation of effort is whether the measured effects reflect changes in judgments or perception. To put the question simply: do we really “see” the hill as steeper if we are tired or do we simply report it as such? We know from other areas of judgment and decision-making that framing can have a strong influence on decisions. In moral reasoning, seemingly unimportant phrasing differences can sway participants’ decisions (Petrinovich and O’Neill, 1996; Cao et al., 2017) as is the case in decision-making (Wang and Johnston, 1995; LeBoeuf and Shafir, 2003). An interesting approach for future study could be to try to manipulate only the type and framing of the cover story to see whether the effects on estimation would be as pronounced as the framing effects reported in the decision-making literature. Furthermore, given the social nature of experiments (Roepstorff and Frith, 2004), another interesting direction would be to investigate whether the way information about the task is structured and communicated influences participants’ estimates.

In conclusion, our findings raise challenges for the interpretation of effects of effort on perceptual estimates and suggest that how participants interpret the task might play a strong role in modulating their estimates of the physical environment.

## Data Availability Statement

The original contributions presented in the study are included in the article/Supplementary Material, further inquiries can be directed to the corresponding author/s.

## Ethics Statement

The studies involving human participants were reviewed and approved by the United Ethical Review Committee for Research in Psychology. The patients/participants provided their written informed consent to participate in this study.

## Author Contributions

VK and NS developed the tasks together. VK collected, analyzed, and interpreted the data under the supervision of NS. Both authors worked on the manuscript.

## Conflict of Interest

The authors declare that the research was conducted in the absence of any commercial or financial relationships that could be construed as a potential conflict of interest. The handling editor declared a past co-authorship with one of the authors NS.

## Footnotes

1 The real mean distance was 7.5 m. Participants were the most precise in Experiment 2 (*M* = 7.74) in which they traversed distances but there was no significant effect of effort. This was followed by Experiment 4 (*M* = 8.59) where participants were stationary and there was a significant effect of effort.
