# Supplementary material for: Task Construal Influences Estimations of the Environment
Source: Front Hum Neurosci. 2021 Jun 10;15:625193. doi: 10.3389/fnhum.2021.625193 (PMC8223064; doi:10.3389/fnhum.2021.625193)
Supplement: Supplementary file 1 [file Data_Sheet_1.docx]

Supplementary Materials

**MEANS AND STANDARD DEVIATIONS PER CONDITION (Exp. 1-4)**

Experiment 1: Verbal estimates

|  | low shelf | medium shelf | high shelf |
| --- | --- | --- | --- |
| light weight | 99 (17) | 126 (19) | 153 (10) |
| medium weight | 96 (21) | 124 (17) | 150 (14) |
| heavy weight | 97 (21) | 123 (20) | 151 (12) |

Table 1. Mean verbal height estimates (standard deviations) per condition

Experiment 1: Non-verbal estimates

|  | low shelf | medium shelf | high shelf |
| --- | --- | --- | --- |
| light weight | 82 (21) | 96 (22) | 112 (23) |
| medium weight | 81 (20) | 97 (24) | 112 (26) |
| heavy weight | 83 (19) | 97 (23) | 109 (27) |

Table 2. Mean non-verbal height estimates (standard deviations) per condition

Experiment 2: Verbal estimates

|  | **MOVEMENT** | |
| --- | --- | --- |
| **DISTANCE** | **Walking** | **Hopping** |
| **1** | 0.95 (0.43) | 0.95 (0.37) |
| **2** | 1.92 (0.63) | 1.7 (0.57) |
| **3** | 3.14 (1.36) | 2.64 (1.19) |
| **4** | 3.72 (1.17) | 3.8 (1.4) |
| **5** | 5.2 (1.76) | 5.07 (2.12) |
| **6** | 6.43 (1.69) | 6.1 (2.90) |
| **7** | 7.03 (2.18) | 7.08 (2.27) |
| **8** | 8.97 (4.12) | 8.24 (3.3) |
| **9** | 9.83 (3.47) | 9.82 (3.98) |
| **10** | 11.09 (4.75) | 10.39 (3.55) |
| **11** | 11.74 (4.68) | 10.95 (4.02) |
| **12** | 12.09 (4.41) | 12.44 (4.34) |
| **13** | 13.78 (4.62) | 13.01 (4.33) |
| **14** | 14.39 (5.01) | 14.16 (4.72) |

Table 3. Mean (SDs) verbal estimates of distance per condition. Distances are expressed in meters.

EXPERIMENT 2: Time

|  | **MOVEMENT** | |
| --- | --- | --- |
| **DISTANCE** | **Walking** | **Hopping** |
| **1** | 2.19 (2.09) | 1.95 (1.01) |
| **2** | 2.77 (1.63) | 2.18 (0.46) |
| **3** | 3.05 (0.6) | 2.81 (0.8) |
| **4** | 3.64 (0.94) | 3.21 (0.59) |
| **5** | 4.59 (1.14) | 3.85 (0.82) |
| **6** | 4.62 (0.95) | 4.66 (1.42) |
| **7** | 5.56 (0.78) | 4.7 (0.81) |
| **8** | 6.41 (1.22) | 5.19 (1.06) |
| **9** | 6.28 (0.85) | 5.89 (1.15) |
| **10** | 7.44 (1.78) | 5.98 (1.23) |
| **11** | 7.92 (1.09) | 7.27 (1.75) |
| **12** | 7.84 (1.91) | 7.41 (1.24) |
| **13** | 9.19 (1.09) | 7.87 (1.53) |
| **14** | 9.52 (1.51) | 8.87 (2.12) |

Table 4. Mean time (SDs) in seconds participants took to cross each distance.

EXPERIMENT 3: Verbal estimates

|  | **BACKPACK** | |
| --- | --- | --- |
| **DISTANCE** | **Empty** | **Full** |
| **1** | 1.06 (0.46) | 0.86 (0.39) |
| **2** | 1.94 (0.81) | 2.58 (2.79) |
| **3** | 3.16 (1.3) | 3.17 (1.38) |
| **4** | 4.31 (1.69) | 4.32 (1.75) |
| **5** | 5.29 (1.93) | 5.68 (2.42) |
| **6** | 6.63 (2.86) | 7.09 (3.27) |
| **7** | 8.29 (3.41) | 8.31 (3.64) |
| **8** | 9.4 (4.17) | 9.23 (3.45) |
| **9** | 10.91 (5.04) | 10.83 (4.71) |
| **10** | 11.07 (4.61) | 11.85 (4.62) |
| **11** | 12.76 (5.49) | 13.15 (5.36) |
| **12** | 14.09 (6.38) | 14.09 (6.13) |
| **13** | 14.83 (6.15) | 13.89 (6.63) |
| **14** | 16.28 (7.07) | 15.77 (6.9) |

Table 5. Mean (SDs) verbal estimates of distance per condition. Distances are expressed in meters.

EXPERIMENT 3: Time

|  | **BACKPACK** | |
| --- | --- | --- |
| **DISTANCE** | **Empty** | **Full** |
| **1** | 1.59 (0.31) | 1.71 (0.41) |
| **2** | 2.08 (0.41) | 2.8 (1.72) |
| **3** | 2.85 (0.42) | 3.19 (0.37) |
| **4** | 3.57 (0.58) | 3.82 (0.68) |
| **5** | 4.27 (0.46) | 4.56 (0.49) |
| **6** | 4.93 (0.61) | 5.2 (0.58) |
| **7** | 5.86 (0.71) | 6.32 (0.84) |
| **8** | 6.48 (0.7) | 6.98 (0.84) |
| **9** | 7.16 (0.71) | 7.81 (0.77) |
| **10** | 8.14 (0.95) | 8.12 (1) |
| **11** | 8.39 (1.83) | 9.12 (0.84) |
| **12** | 9.03 (1.19) | 9.51 (0.87) |
| **13** | 9.9 (1.29) | 10.23 (1.07) |
| **14** | 10.69 (1.16) | 10.95 (1.15) |

**Table 6**. Mean time (SDs) in seconds participants took to cross each distance.

**PARTICIPANTS' PERFORMANCE ANALYSIS**

**EXPERIMENT 1**

Pearson correlation showed that participants' verbal estimates of height were highly correlated with actual shelf height (r(486) = .768, p < .001). The correlation between estimates and actual distances was computed for each participant, converted by a Fisher Z-transformation and a single sample t-test with a test value of 0 was run (t(17) = 14.38, p < 0.001, d = 3.39) which confirmed a good overall performance on the task. The same analyses were performed for non-verbal responses resulting in a high Pearson correlation (r(486) = .453, p < .001), additionally confirmed by a t-test (t(17) = 6.72, p < 0.001, d = 1.58).

**EXPERIMENTS 2-4**

The same analyses were conducted for participants' distance estimates in Experiments 2-4 showing a high correlation between estimates and actual distances. For Experiment 2, the analyses revealed a high Pearson correlation (r(476) = .805, p < .001) and a significant single sample t-test (t(16) = 28.29, p < 0.001, d = 6.86). The same was the case for Experiment 3 (r(476) = .75, p < .001 and t(19) = 19.01, p < 0.001, d = 4.26) and Experiment 4 (r(532) = .799, p < .001 and t(18) = 24.75, p < 0.001, d = 5.68).

**BAYESIAN ANALYSES (EXPERIMENTS 1-4)**

**EXPERIMENT 1**

In order to test whether the data support the null hypothesis that weight does not influence judgment estimates, the data were broken down by weight and a series of Bayesian paired-samples t-tests were conducted using a built-in function of JASP. Before the analyses the data were checked to confirm that there were no outliers (see Figure 4.). Shapiro-Wilks tests confirmed that the normality assumption was not violated for comparisons between light and medium (p = 0.32), heavy and light (p = 0.44) and heavy and medium weights (p = 0.8) for verbal responses. Furthermore, no violation was detected for non-verbal responses for comparisons between light and medium (p = 0.45), heavy and light (p = 0.53) and heavy and meidum weights (p = 0.52).


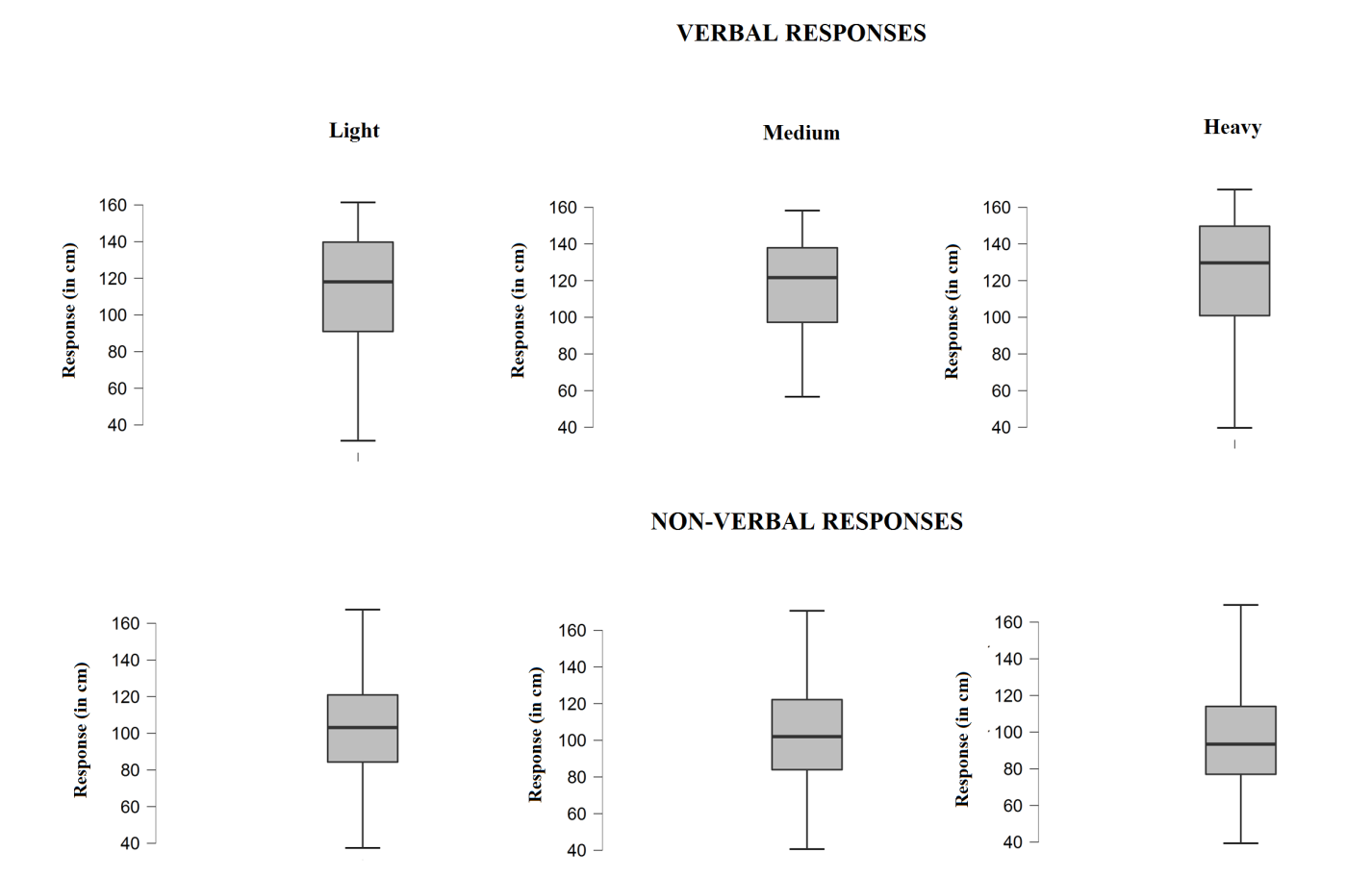


Figure 1. Boxplots of verbal and non-verbal responses for each weight.

In all tests, H_0_ stated that the effect size is δ = 0 while H_1_ assigned effect size a Cauchy prior centered on 0 with the interquartile range of r = .707. For verbal responses, comparing the influence of heavy and medium weight (see Figure 4) on participants’ estimates, showed strong support for the null hypothesis (BF_01_ = 11.32). Comparisons between heavy weight and light weight received moderate support (BF_01_= 7.82) as did those of light and no mediumt (BF_01_= 7.26). For non-verbal responses, the null hypothesis was strongly supported in all three comparisons showing BF_01_ ≈ 11 (see Figure 4 for exact values).


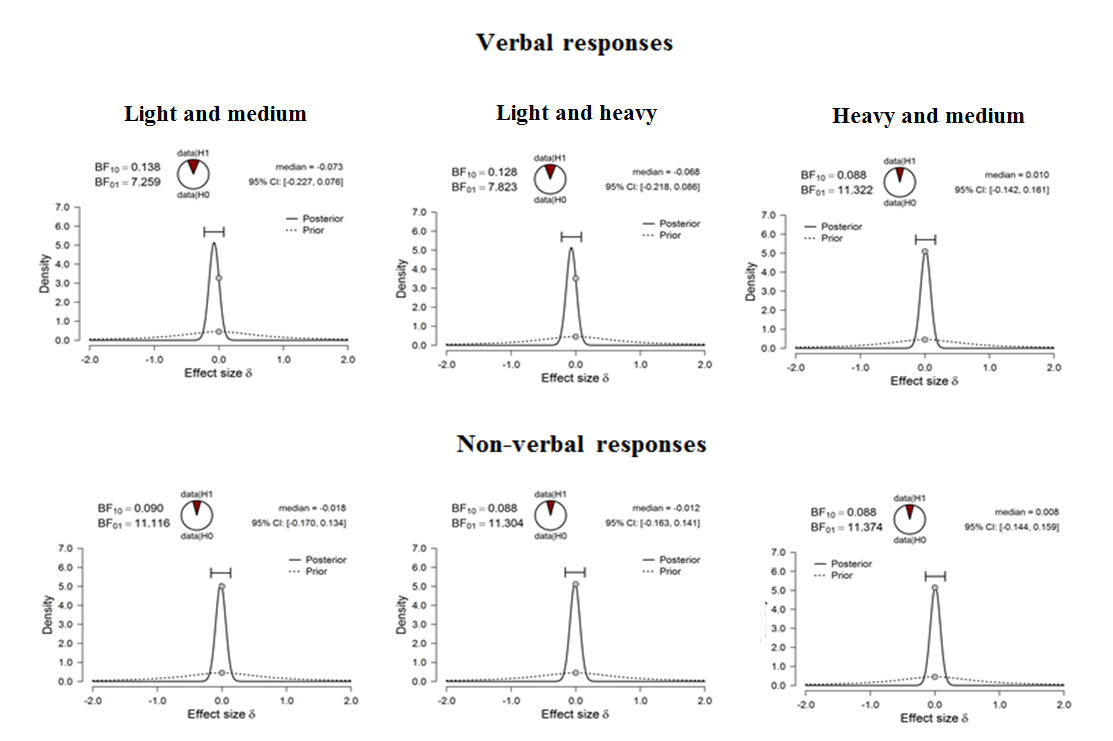


Figure 2. Pairwise comparisons of verbal and non-verbal estimates in different weight conditions. Horizontal bars represent 95% credibility intervals.


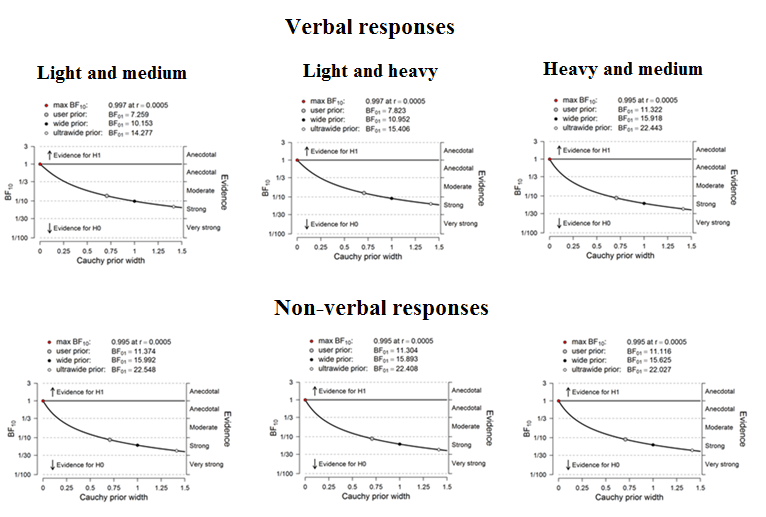


Figure 3. Comparing the prior set in the experiment (r = 0.707) to a wide (r = 1) and ultrawide prior (r = 1.41).

A Bayes factor robustness analysis was conducted using a number of alternative priors (see Figure 5) to see if the chosen prior was a reasonable one. For both verbal and non-verbal responses, it showed that different priors would not change the qualitative conclusion of the data supporting the null hypothesis. Under wider priors than the ones chosen for the analysis, the Bayes factor would give stronger support for the null. Taking into account the robustness and magnitude of the Bayes factor, the analysis shows that the data moderately to strongly suggest that weight did not have an effect on verbal and non-verbal height estimates.

**EXPERIMENT 2**

To establish if the data supports that the hopping and walking did not influence distance estimates differently, a Bayesian paired samples t-test was performed. Before the analysis, the data was checked for outliers (see Figure 7) and the normality assumption was confirmed with a Shapiro-Wilk test (p = 0.51) As in the analysis in Experiment 1, the prior was Cauchy (0, .707).


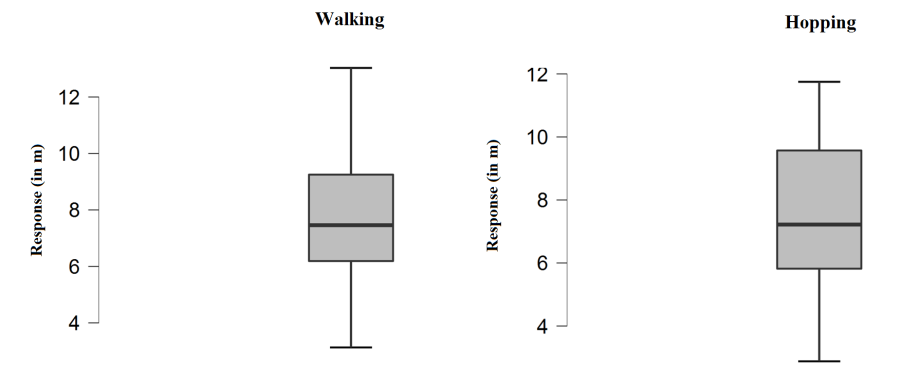


Figure 4. Boxplots of verbal and non-verbal responses for the two effort manipulations.


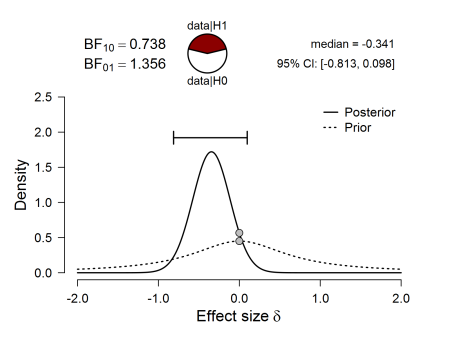

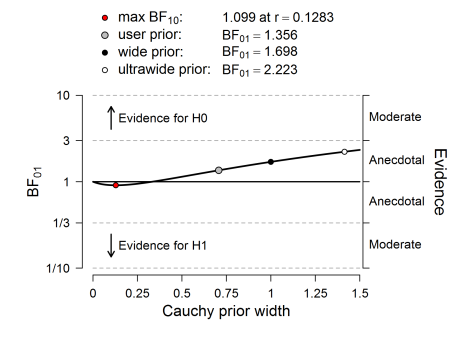


Figure 5. Bayes factor and robustness check for influence of hopping and walking on distance estimates.

The analysis revealed weak evidence that the data support the null hypothesis. A robustness check further suggested that while wider priors would have increased the relative evidence for H_0_, it would still remain anecdotal.

**EXPERIMENT 3**

A Bayesian equivalent of a paired-samples t-test was conducted using a built-in function in JASP. The analysis used a Cauchy prior (0, .707). Shapiro-Wilk test confirmed that the normality assumption was not violated (p = 0.9). Removing the single outlier did not change the results so it was kept in the analysis for the sake of completeness.


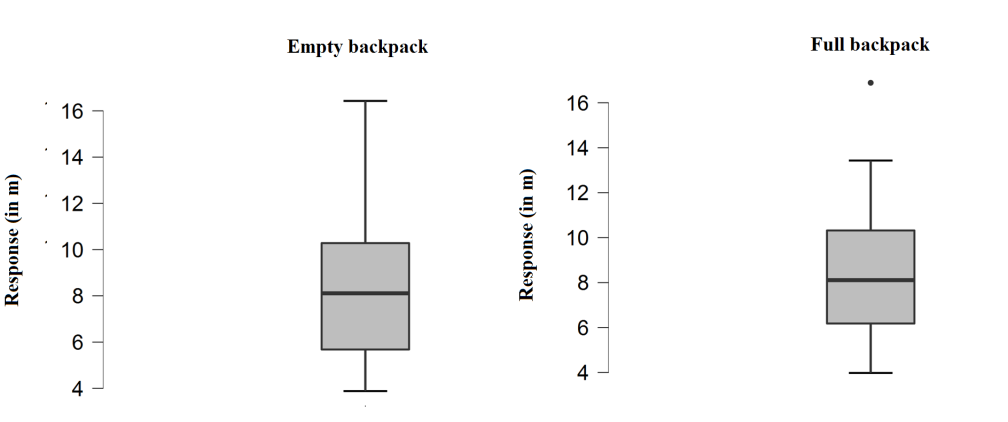


Figure 6. Boxplots of verbal and non-verbal responses for the two effort manipulations.


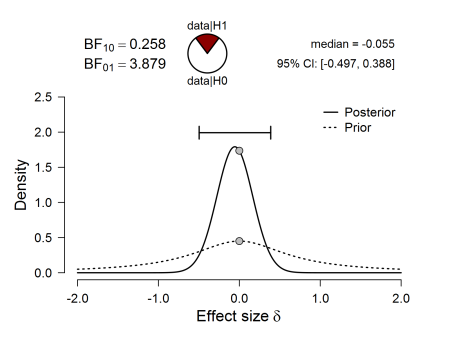

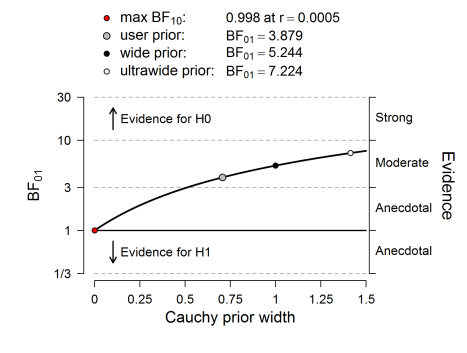


Figure 7. Bayes factor and robustness check for influence of hopping and walking on distance estimates.

Results suggested moderate support for the null (BF_01_ = 3.88). Robustness analysis showed that wider priors would provide stronger evidence for H_0_ but support would remain in the moderate range.

**EXPERIMENT 4**

A Bayesian equivalent of a paired-samples t-test was conducted using a built-in function in JASP. The analysis used a Cauchy prior (0, .707). Shapiro-Wilk test confirmed that the normality assumption was not violated (p = 0.99).


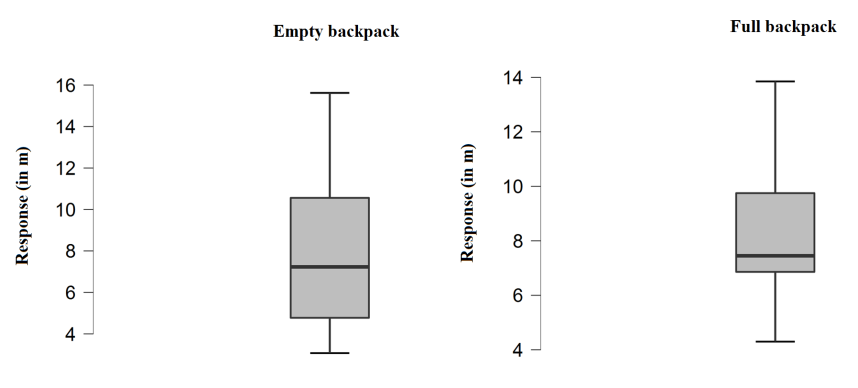


Figure 8. Boxplots of verbal and non-verbal responses for the two effort manipulations.

#####
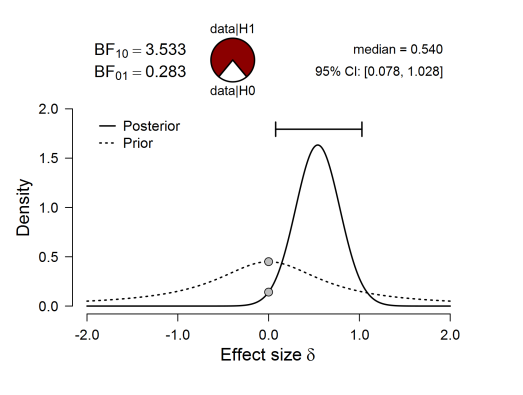

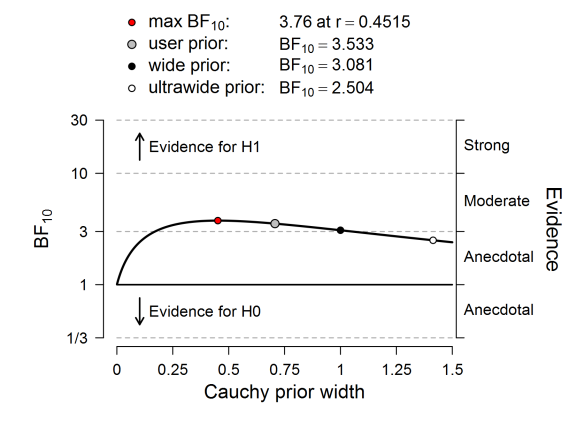


The analysis revealed moderate support for the alternative hypothesis. A robustness check further suggested that while wider priors would have decreased the evidence for H_0_ with evidence becoming anecdotal if r = √2.

**ANALYSIS OF EFFECTS OF LEARNING AND MOVEMENT**

One key difference between Experiment 4 and the previous two experiments should be addressed. In Experiments 2 and 3, participants traversed the distance rather than standing in place. It is possible that moving across the distance served as a strong cue for estimation and therefore participants did not have to resort to effort-based heuristics. To account for this possibility, data from this experiment as well as the two previous ones were re-analyzed to look at estimates over time. Trials were split into two bins: the first 14 and the second 14 trials. If moving across the distance informed estimates, then participants’ estimates should improve over the course of the experiment. A paired-sample t-test from the data of Experiment 2 (walking/hopping) did not show significant differences t(17) = -0.116, p = 0.909). The same analysis for Experiment 3 (walking with empty/heavy backpack) revealed a significant difference t(17) = -3.03, p = 0.008, d = -0.73). Participants judged the distances closer to the actual mean distance of 7.5 in the first half of the experiment (M = 8.37, SD = 3.29) than in the second half (M = 8.84, SD = 3.47). This result shows that estimates did not improve over the course of the experiment. Additional paired-samples t-tests showed that participants’ performance did not improve during the second part of Experiment 1 (t(17) = -0.278, p = 0.784) and Experiment 4 (t(18) = 1.47, p = 0.16).
